# Contouring practices and artefact management within a synthetic CT-based radiotherapy workflow for the central nervous system

**DOI:** 10.1186/s13014-024-02422-9

**Published:** 2024-02-29

**Authors:** Elia Rossi, Sevgi Emin, Michael Gubanski, Giovanna Gagliardi, Mattias Hedman, Fernanda Villegas

**Affiliations:** 1 Department of Radiation Oncology, Karolinska University Hospital, Solna, Sweden; 2 Radiotherapy Physics and Engineering, Medical Radiation Physics and Nuclear Medicine, Karolinska University Hospital, Solna, Sweden; 3 Department of Oncology-Pathology, Karolinska Institutet, Solna, Sweden

**Keywords:** MR-only, Synthetic-CT, Radiation therapy, Contouring process, Metal artefacts

## Abstract

**Background:**

The incorporation of magnetic resonance (MR) imaging in radiotherapy (RT) workflows improves contouring precision, yet it introduces geometrical uncertainties when registered with computed tomography (CT) scans. Synthetic CT (sCT) images could minimize these uncertainties and streamline the RT workflow. This study aims to compare the contouring capabilities of sCT images with conventional CT-based/MR-assisted RT workflows, with an emphasis on managing artefacts caused by surgical fixation devices (SFDs).

**Methods:**

The study comprised a commissioning cohort of 100 patients with cranial tumors treated using a conventional CT-based/MR-assisted RT workflow and a validation cohort of 30 patients with grade IV glioblastomas treated using an MR-only workflow. A CE-marked artificial-intelligence-based sCT product was utilized. The delineation accuracy comparison was performed using dice similarity coefficient (DSC) and average Hausdorff distance (AHD). Artefacts within the commissioning cohort were visually inspected, classified and an estimation of thickness was derived using Hausdorff distance (HD). For the validation cohort, boolean operators were used to extract artefact volumes adjacent to the target and contrasted to the planning treatment volume.

**Results:**

The combination of high DSC (0.94) and low AHD (0.04 mm) indicates equal target delineation capacity between sCT images and conventional CT scans. However, the results for organs at risk delineation were less consistent, likely because of voxel size differences between sCT images and CT scans and absence of standardized delineation routines. Artefacts observed in sCT images appeared as enhancements of cranial bone. When close to the target, they could affect its definition. Therefore, in the validation cohort the clinical target volume (CTV) was expanded towards the bone by 3.5 mm, as estimated by HD analysis. Subsequent analysis on cone-beam CT scans showed that the CTV adjustment was enough to provide acceptable target coverage.

**Conclusion:**

The tested sCT product performed on par with conventional CT in terms of contouring capability. Additionally, this study provides both the first comprehensive classification of metal artefacts on a sCT product and a novel method to assess the clinical impact of artefacts caused by SFDs on target delineation. This methodology encourages similar analysis for other sCT products.

## Background

Dedicated magnetic resonance (MR) scanners for radiotherapy (RT) workflows have increased in the past decades, proving to be beneficial as MR-imaging allows a more accurate contouring of tumour tissue and organs at risk [1, 2]. A computed tomography (CT) scan is still essential in the RT workflow because it provides the electron density (ED) information needed for dose simulation. An MR-assisted RT workflow inevitably includes a registration process whereby both image modalities are fused together to permit the propagation of the contours, delineated on the MR images, onto the CT scans prior to dose calculation. This registration process, however, introduces geometrical uncertainties that are accounted for as an expansion of the planning target volume (PTV) [3].

Advances in computation technology and MR imaging have opened the possibility to generate CT-like images directly from MR sequences. In an MR-only RT workflow, these so-called synthetic CT (sCT) images substitute the planning CT scans. This workflow not only eliminates the registration uncertainties but also simplifies the clinical workload as only one image examination, namely MR imaging, is needed for RT planning [4–6]. An MR-only RT workflow can be enabled by a two-step implementation process: firstly with a commissioning phase, to test whether the sCT image can perform equally as a CT scan in a clinical environment, and secondly with a validation phase, to develop new workflow guidelines as part of a quality assurance (QA) management program that would oversee the safe usage of the sCT images in RT.

One key point of the commissioning process is to assess the image quality of the sCT image in comparison to the planning CT scan. As sCT images are generated by computer algorithms, deviations from CT scans are expected. The magnitude of these deviations decreases with increasing complexity of the algorithm used, i.e., from the simplest bulk density method to machine learning (ML) methods with varying layers of complexity.

Amongst the most common problematic tissues for sCT image generation is bone due to its complex anatomical structure, varying density patterns, heterogeneous composition, and distinct imaging characteristics of the paired CT scans and MR images used for training the algorithm [7]. The presence of metal implants, dental fillings and anatomical variations caused by pathologies or surgeries further complicates the training process. Another common complication regards air-filled structures, such as sinuses and air cavities, due to their low-density nature and the resulting sharp transitions of attenuation values between surrounding tissue [8]. These challenges can lead to the presence of artefacts in the sCT images. Therefore, understanding artefact sources and characteristics is essential for implementing effective strategies to detect and address them, ultimately reducing their impact on treatment plans.

This work aimed at analysing various aspects of the contouring capability of a commercial sCT product compared to a conventional CT-based/MR-assisted RT workflow. Local consensus recommendations on contouring and image QA criteria were developed with particular focus on handling of artefacts on sCT images. As a result, Karolinska University Hospital has successfully implemented this RT workflow for glioblastoma grade IV patients.

## Methods

### Patient cohort

Between January 2021 and February 2023, the implementation of an MR-only workflow for central nervous system (CNS) patients was carried out in two phases: commissioning and validation, as outlined in Table 1. The study was approved by the Swedish Ethical Review Authority (dnr 2019-06404). For the commissioning phase, a prospective evaluation of 112 patients diagnosed with CNS tumors who were referred to Karolinska University Hospital for treatment was conducted. The complete list of diagnoses and fractionation schemes is included in Table 2. Patients above 18 years of age with no contraindications for MR imaging and who provided informed oral and written consent were included. All patients received standard CT-based-planned treatment either with photons or with protons. The validation phase consisted of 30 patients diagnosed with grade IV glioblastoma who received photon treatment following the same fractionation schemes outlined in Table 2 and met the identical inclusion criteria. Tumour classification was carried out using the World Health Organization (WHO) 2016 classification of glioma.

**Table 1:** Implementation process for MR-only workflow for radiotherapy of CNS tumours

	Phase 1	Phase 2
Objective	Commissioning	Validation
Simulation imaging	CT and MRI	CT and MRI
MR-CT image registration	Rigid registration based on bone anatomy	Rigid registration based on bone anatomy
Delineation	Bones, Body and Brain on CT. Target and OARs on MRI	Bones, Body and Brain on sCT. Target and OARs on MRI
Dose optimisation	Performed on CT	Performed on sCT
Patient position verification	CT-CBCT	sCT-CBCT
Patient cohort	112 CNS cancer patients	30 glioblastoma g.IV patients

**Table 2:** List of diagnosis and their relative treatment plans

Diagnosis		Plan	Fractionation (Gy)	CTV margin (mm)
G. I-II (22)	OG. (12)	Ph. (1)	$2\times 30$	15*
		Pr. (11)	$1.8\times 28$-30-33	
	AC. (10)	Ph. (3)	$3.4\times 10; 2\times 30$	
		Pr. (7)	$1.8\times 28$-30	
Anaplastic G. II (7)	Anaplastic OG. (1)	Pr. (1)	$1.8\times 33$	20*
	Anaplastic AC. (6)	Ph. (4)	$2\times 30$	
		Pr. (2)	$1.8\times 33$	
GB. III-IV (51)	AC. III (2)	Ph. (2)	$3.5\times 10; 2.67\times 15$	20*
	OG. III (2)	Ph. (1)	$2.67\times 15$	
		Pr. (1)	$1.8\times 33$	
	GB. IV (47)	Ph. (46)	3.4–$3.5\times 10; 2.67\times 15; 2\times 30$	
		Pr. (1)	$1.8\times 28$	
Menigiomas (8)	Meningioma I (6)	Pr. (6)	$1.8\times 28$-30-33;	2–5*
	Meningioma II (1)	Pr. (1)	$1.8\times 30$	5–10*
	HmngP. II-III (1)	Ph. (1)	$2\times 30$	**
Benign Neoplasms (2)	Craniopharingioma (1)	Pr. (1)	$1.8\times 28$	1.5*
	Schwannoma (1)	Pr. (1)	$1.8\times 28$	**
Metastasis (9)		Ph. (9)	$6\times 5$	CTV = GTV
Neuroendocrine T. (1)	Embryonic T. (1)	Ph. (1)	$6\times 5$	**

The number of patients per diagnosis is enclosed in parenthesys. Most CTV margin (marked with *) have a specific set value but can vary depending on tumour extention; for others (marked with **) margin is adapted in each case

*Ph* Photon plan, *Pr* Proton plan, *G* Gliomas, *OG* Oligodendrogliomas, *AC* Astrocytoma, *GB* Glioblastomas, *HmngP* Hemangiopericytoma, *T* Tumours

### Image acquisition and sCT generation

Planning CT scans without contrast medium were obtained during both the commissioning and validation phase from a Siemens Somatom Definition AS. Images were acquired with a voxel size of 1.00/1.00/2.00 (mm) without metal-artefact reduction techniques. For MR images, a Philips Ingenia 3.0T system (Koninklijke Philips N.V., Best, The Netherlands) was used to acquire T1-weighted with contrast medium Gadolinium (MRI T1 Gd+), T2-weighted and fluid-attenuated inversion recovery (FLAIR) images, required for contouring with a total acquisition time of 17 min and 14 s. A dedicated 3D fast field echo (FFE) mDIXON sequence with short echo time and high bandwidth was also added to the MR protocol for sCT generation, taking an additional 4 min and 20 s, bringing the total protocol time to just over 22 min. The artificial intelligence (AI)-based Philips magnetic resonance for calculating attenuation map (MRCAT) brain algorithm (Philips Oy, Vantaa, Finland), trained with a convolutional neural network [9], was utilized for the generation of sCT images. This image processing occurs in the background, and as such, it does not contribute any additional time to the MR protocol. The voxel size of the sCT images was 0.68/0.68/1.00 (mm). CT scans and MR images were ideally obtained on the same day to minimize inter-scan positioning errors. Both scanners were equipped with flat tabletops and laser positioning systems identical to those used in the treatment room. The fixation protocol included the use of neck support, laser positioning markers and a three point head thermal mask to immobilize the patient’s head during each treatment session. The planning CT scans and MR images for each patient were transferred to Eclipse Treatment Planning System (TPS) (v. 16.01.10, Varian Medical Systems, Inc), and a rigid registration was performed between the two sets. The key difference between patients in the commissioning phase and those in the validation phase is that, for the latter, the CT scans were acquired after the MR images and were used for QA purposes.

### Image quality of sCT images

As part of the commissioning phase, an image QA was conducted by visually comparing the sCT images to their corresponding CT scans. Of particular interest was the handling of metal implants by the MRCAT algorithm. In MR images, metallic implants cause a loss of signal resulting in a blackout around the position of the implant [10, 11], while streak artefacts are typically seen in CT scans. The use of dedicated metal reduction reconstruction algorithms [12] is encouraged, especially when the target volume is found in the vicinity of the metal implant [13, 14]. On the other hand, the appearance of these kind of artefacts in the tested sCT images was unknown, hence identification and classification was done. Two kinds of metal implants were identified in the commissioning phase cohort:

**Fig. 1:**
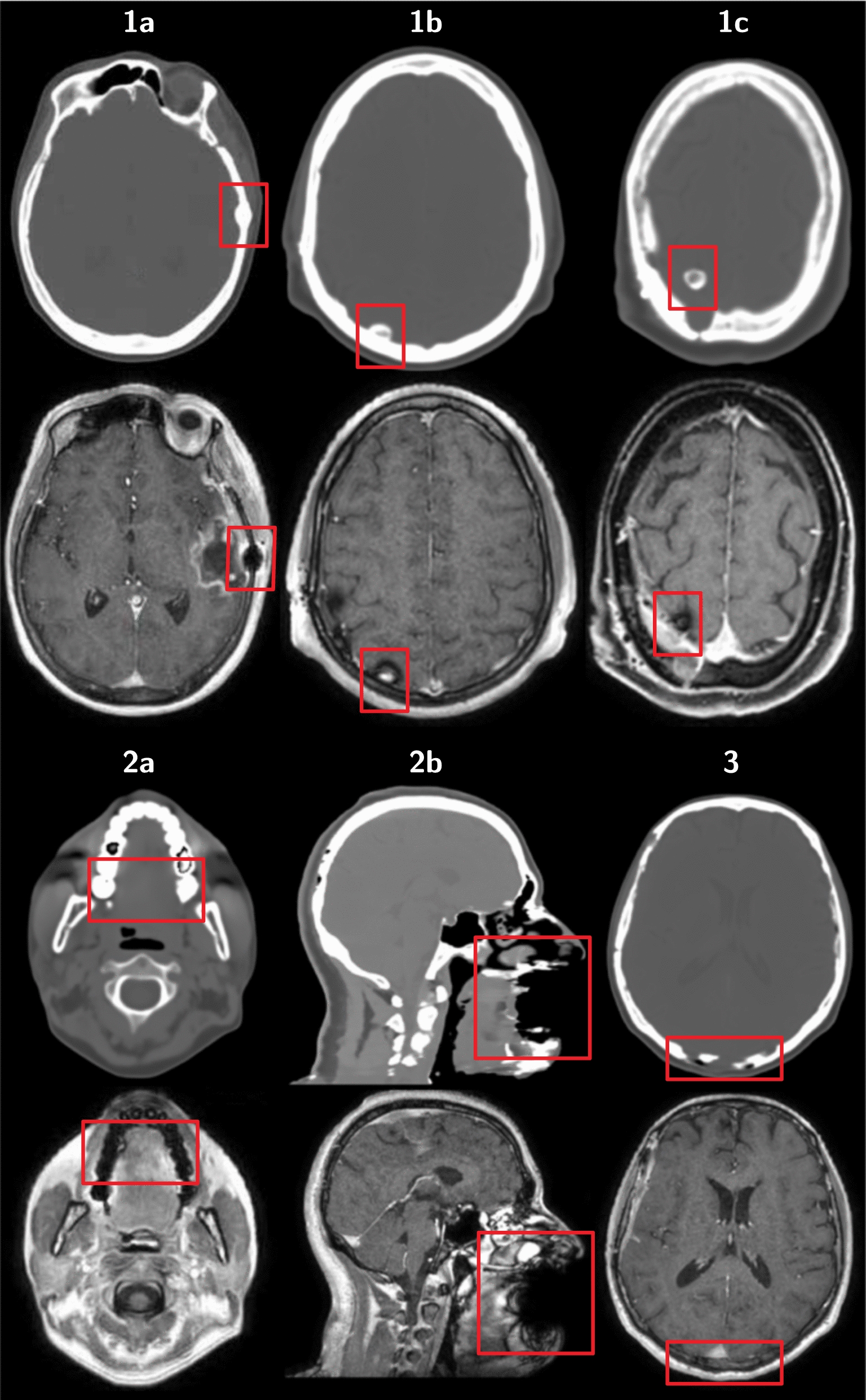
Classification of artefacts (red boxes) on sCT (above) and the relative source MR mDIXON in-phase sequence (below). Type 1, known as SFD artefacts, are sub-classified based on their position relative to bones (type 1a-b-c). Type 2a-b artefacts are associated with dental implants. Type 3 artefacts are characterised by a bone gap

**Fig. 2:**
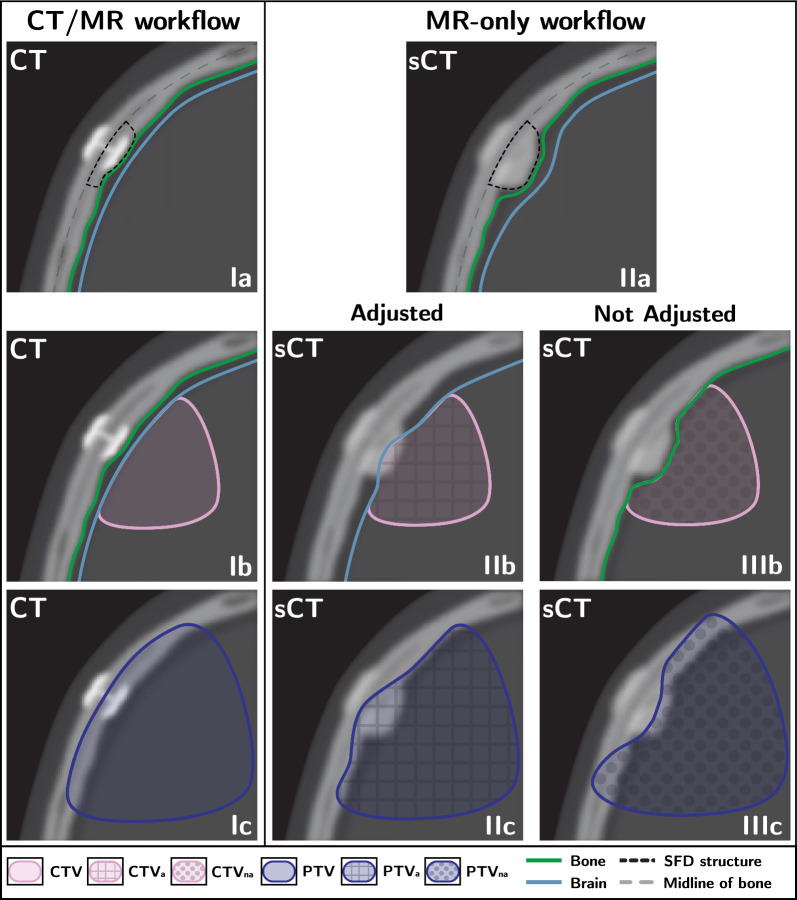
Changes in the way CTV is cropped between the CT-based/MR-assisted workflow (Ib) and the MR-only workflow both with adjustments (IIb) and without(IIIb). The effect on the PTV is shown in Ic, IIc and IIIc

*Clamp-based surgical bone fixation devices (SFD)* (type 1 on Fig. 1): Since most CNS patients who undergo radiotherapy have had surgery, SFD used for bone flap fixation are frequently present in the images used for treatment planning. The void in the MR images left by these SFDs is interpreted as bone tissue by the sCT algorithm, resulting in a visible expansion of the skull when compared to the CT scans (see Fig. 2-Ia and IIa). In this work, we will define the volume that should have been interpreted as brain tissue by the sCT algorithm but instead resulted in an expansion of the bone, as SFD artefact.

As SFDs are rigid structures, an optimal conformity to the curvature of the bone is only achieved when the center axis of the SFD is perpendicular to the bone (illustrated in the Appendix Fig. 7). In such situations the signal loss sits on both sides of the skull, creating a cylindrical-shaped volume of bone in the sCT image that has been sub-classified as SFD artefact type 1a. In case the SFD’s position is more cranial, its orientation deviates causing more signal loss towards the brain tissue in the initial slices, before transitioning again into a SFD artefact type 1a. However, since the artefact, in these instances, is initially visible only on the inside part of the skull, it has been sub-classified as type 1b. In the most cranial slices this effect is even more noticeable causing the entire artefact to reside in the brain matter, detached from the bone, sub-classified as type 1c.

*Dental implants* (type 2 on Fig. 1): Unlike streak artefacts typical of CT scans, dental implants are rendered in the sCT images as cavities within the affected teeth with a steep Hounsfield unit (HU) gradient (type 2a). In 10 cases, the quantity and proximity of dental implants had a noticeable effect on a larger portion of the image, creating a severe loss of information around the mouth and jaw as seen in type 2b. However, this is usually not clinically significant for cranial tumors since the treatment volume is sufficiently far from the treated area.

An additional type of artefact not related to metal implants was observed in the form of missing bone:

*Bone gaps* (type 3 on Fig. 1): These artefacts are regions of the skull that appear as bone resection sites in the sCT images, despite the clear presence of bone in all MR images. The identified bone gaps presented various widths and lengths with no apparent connection to skull movement during the MR session. Although not part of this work, an analysis of the bone gap sizes in the validation cohort was performed to establish an acceptance criteria for sCT image QA.

### CT-based/MR-assisted contouring workflow

Target and organs at risk (OARs) contours were delineated by different radiation oncologists in a CT-based/MR-assisted workflow, and the contours were peer-reviewed by a team of experts prior to treatment. In this blended workflow, structures are identified using MR images and then transferred to the CT scan via a rigid registration to allow for dose calculation.

The clinical target volume (CTV) and PTV were defined according to in-house guidelines based on the relevant literature for each tumor type. The CTV was cropped to the brain and other anatomical barriers depending on the tumor’s location. The PTV was generated by adding a 3 mm isotropic margin to the CTV (see Fig. 2-Ia, Ib and Ic).

The Eclipse TPS offers three different tools for automatic contouring of certain organs such as body, brain and bone [15, 16]. The Search Body tool, used only for generation of the body structure, starts by applying a pre-defined HU threshold to which post processing filters are applied. The Flood Fill tool iteratively fills a connected region with similar reference values when a specific area of the image is selected; a goodness value defines the range of HU to be considered similar. Finally the Segmentation Wizard tool utilises Flood Fill with low goodness value to initially segment the main volume according to parameters tuned for specific organs such as brain and bone. It then clears all planes that are too far from the segmented region before dilating the resulting volume with a stronger filling parameter. In this workflow, the body is contoured using the Search Body tool with a threshold of -350 HU, while the brain and bone structures are contoured by applying the Segmentation Wizard tool. When needed, the brain volume was manually corrected only in the region close to the target.

Relevant contouring atlases were used to delineate the other OARs, although radio-oncologists were authorized to make their own annotations. Unfortunately, the specific MR sequence used for delineation of the OARs was not recorded.

### MR-only contouring workflow

All MR-only contours during the commissioning phase were delineated by a single radio-oncologist using a Wacom Cintiq tablet and pen in Eclipse and were peer-reviewed by a team of experienced radio-oncologists. For the commissioning phase, the CTV and PTV were defined using the same in-house guidelines previously mentioned. A new internal guideline adapted from the European Particle Therapy Network (EPTN) consensus for the European Society for Radiotherapy and Oncology (ESTRO) [17, 18] was implemented for the delineation of the OARs.

*Body:* Automatically generated on sCT images by Search Body tool with a threshold of -850 HU. *Brain:* Automatically segmented on sCT images with FloodFill tool and manually adjusted around the target when needed. *Bones:* Automatically contoured on sCT images with Segmentation Wizard tool. *Brainstem:* Contoured on MRI T1 Gd+ starting from the tip of Dens (C2) and extending upwards until the confluence of aqueducts with the 3rd ventricle. Contrary to EPTN’s recommendation, brainstem is delineated as one single volume. *Chiasma:* Contoured on MRI T1 Gd+ starting from one slice below the separation of optic nerves and extending up to one slice above separation point of optical tracts. *Optic nerve:* Delineated on MRI T1 Gd+ from optic chiasma to the posterior edge of the eyeball. *Cochlea:* Contoured on MRI T2 with particular attention paid to exclude semicircular canals and facial canal. *Hippocampus:* Contoured on MRI T1 Gd+ following the temporal horn of the lateral ventricle as lateral border. Contrary to EPTN’s recommendation, hippocampus is delineated as one single volume. *Pituitary:* Contoured using both MRI T1 Gd+ and T2 for better accuracy. Other than the body, the stalk is also delineated up until the level of optic chiasma.

### Comparison of contours between the two RT workflows

As part of the commissioning process, the contours from the CT-based/MR assisted workflow were compared to those from the MR-only workflow. The CT structure sets (CT-ss) and the MR-only structure sets (sCT-ss) were imported into MICE Toolkit (v.2021.1.0, NONPIMedical AB) for the comparison analysis using dice similarity coefficient (DSC), Hausdorff distance (HD), average Hausdorff distance (AHD) and volume size as metrics. DSC and AHD were used as congruence metrics between structure sets, the first providing volumetric similarity and the second geometrical overlap. In this work we define structures with high congruence values as those where DSC exceeds 0.7 in combination with a AHD less or equal to 1 mm. Conversely, we define structures with low congruence values as those with a DSC lower than 0.7 in combination with AHD higher than 1 mm. Following the approach established by Hu et al., the DSC value of 0.7 was chosen considering the inclusion of small volume structures which DSC is known to penalise [19].

### SFD artefact evaluation

#### Artefact size

Because the SFD artefacts can be found in the vicinity or within the CTV, it was of interest to estimate their thickness to evaluate the necessity of manually extending the target structures to reduce the risk of missing clinically relevant brain tissue near the skull where glioblastoma tumour cells are known to infiltrate [20].

A total of 20 type 1a and 1b SFD artefacts, present in 14 patients from the commissioning cohort, were extracted from paired sCT images and CT scans. On the CT scans, a SFD structure was delineated by taking the volume between the mid line of the skull and the internal head of the SFD as depicted in Fig. 2-Ia. Similarly, the volume containing the SFD and its artefact was delineated on sCT images, making sure that all edges, other than the one extruding into the brain, were coinciding between the image modalities (see Fig. 2-IIa). The difference between these two volumes resulted in the volume of the SFD artefact.

The volumes were exported to MICE Toolkit, where the HD was calculated. By averaging the HD values across the 20 cases, we could determine the average maximum distance between these volumes. This value represents the largest thickness expected from these artefacts.

#### SFD artefact effect on target coverage

At the start of the validation phase it was decided that the brain contour would be manually adjusted to account for the loss of brain tissue coverage in all cases where a SFD artefact was detected within the target. The amount of this adjustment was based on the average HD calculated over the 20 SFD instances selected during the commissioning phase. The procedure for this adjustment is shown in Fig. 2-IIb. Since the CTV is cropped to the brain, the resulting CTV cropped to the adjusted brain is defined as CTV adjusted (CTV$_a$).

An analysis was later conducted on 10 patients from the validation cohort to evaluate the impact of CTV$_a$ on target coverage as observed in the cone-beam CT (CBCT) scans taken before each treatment fraction. First, the corresponding 3 mm PTV (PTV$_a$) and sCT bone volume (B$_{sCT}$) were selected; second, 5 CBCT scans for each patient were chosen, on which Segmentation Wizard was used to automatically contour bone tissue B$_{CBCT}^{i}$, where the index *i* represents each CBCT scan. All structures were transferred to the sCT images using the online registration matrix, so that they all had the same frame of reference when imported into MICE Toolkit.

To obtain the volume of the SFD artefact in relation to each CBCT scan, two additional intersection volumes (IV) were extracted using PTV$_a$ as control VOI, namely:

1 $$\begin{aligned} IV_{CBCT}^{i} = B_{CBCT}^{i} \wedge PTV_a \end{aligned}$$

2 $$\begin{aligned} IV_{sCT} = B_{sCT} \wedge PTV_a \end{aligned}$$

**Fig. 3:**
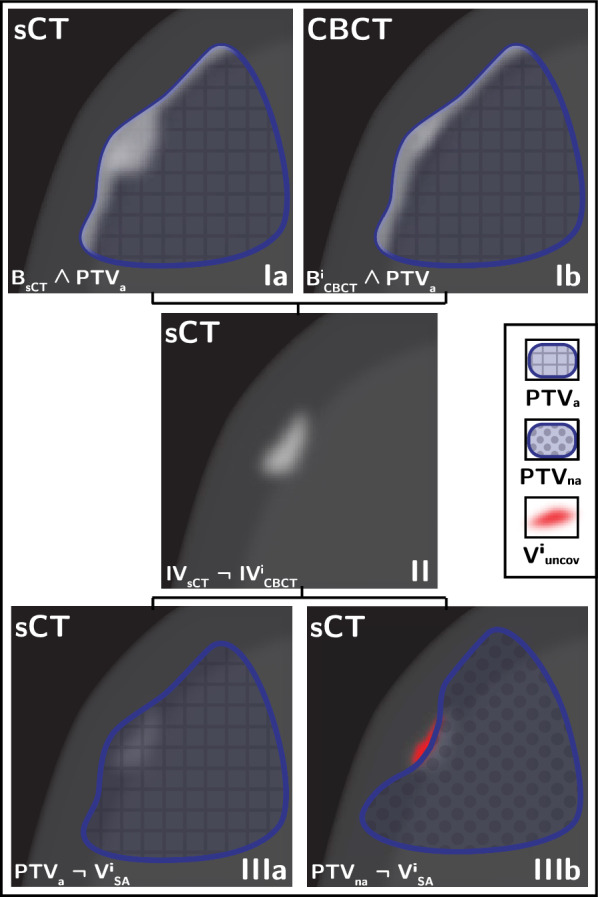
Artefact analysis performed in MICE: intersection volumes (Ia and Ib), volume of the SFD artefact (II) and uncovered volume (IIIa) are shown. Additionally the PTV$_{a}$ coverage is shown in IIIb

Step 1 and 2 are shown in (Fig. 3-Ia and Ib). Thus the volume of the SFD artefact (V$_{A}$) is simply obtained by subtracting (Fig. 3-II):

3 $$\begin{aligned} V_{A}^{i} = IV_{sCT} \lnot IV_{CBCT}^{i} \end{aligned}$$

In contrast to the volume obtained in  Artefact size section, the calculation of V$_{A}$ was performed exclusively using boolean operators within MICE Toolkit, eliminating the need for manual delineation. Furthermore, the purpose of V$_{A}$ was to assess the actual volume rather than just the thickness. With these volumes we could then calculate the volume not covered by the PTV$_{a}$ by simply subtracting (Fig. 3-IIIb):

4 $$\begin{aligned} V_{uncov(a)}^{i} = PTV_{a} \lnot V_{A}^{i} \end{aligned}$$

A second evaluation was performed on the same cohort to determine the impact of an alternative solution which would not involve the manual extension of the brain contour. For each patient, a not-adjusted CTV (CTV$_{na}$) was obtained by duplicating the original CTV$_a$ with the difference that CTV$_{na}$ was cropped against the sCT bone structure B$_{sCT}$ which by default includes the SFD artefact. A corresponding 3 mm PTV was also generated (PTV$_{na}$). All new structures were imported in MICE Toolkit.

Given the V$_{A}$ previously calculated (3) and the new structures, we could then calculate the volume not covered by the PTV$_{na}$ by simply subtracting (Fig. 3-IIIa):

5 $$\begin{aligned} V_{uncov(na)}^{i} = PTV_{na} \lnot V_{A}^{i} \end{aligned}$$

## Results

A total of 12 patients were excluded from the commissioning cohort, 7 (6%) of which did not complete RT due to medical reasons. The remaining 5 (4%) excluded cases refer to failure to generate sCT images. Two reasons were identified as causes: the first being that the disparity of the body contour between the source MR images exceeded the predetermined threshold set by the vendor; and the second was due to incorrect parameters set before image acquisition.

In some cases, the CT-ss did not include all OARs recommended by guidelines as they were considered not relevant based on the tumour’s location. As a result, the comparison was conducted with different amount of structures for each OAR. Additionally, for structures like cochleae, hippocampi and optic nerves, the analysis took values from these structures into account only when both the left and right side were present.

**Fig. 4:**
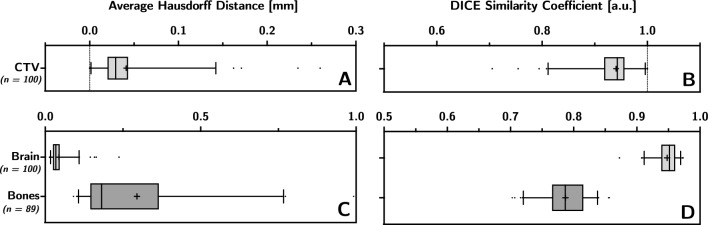
Average Hausdorff distance (**A** and **C**) and dice similarity coefficient (**B** and **D**) of CTV (**A** and **B**), brain and bones (**C** and **D**). | represents the median. + represents the mean

**Fig. 5:**
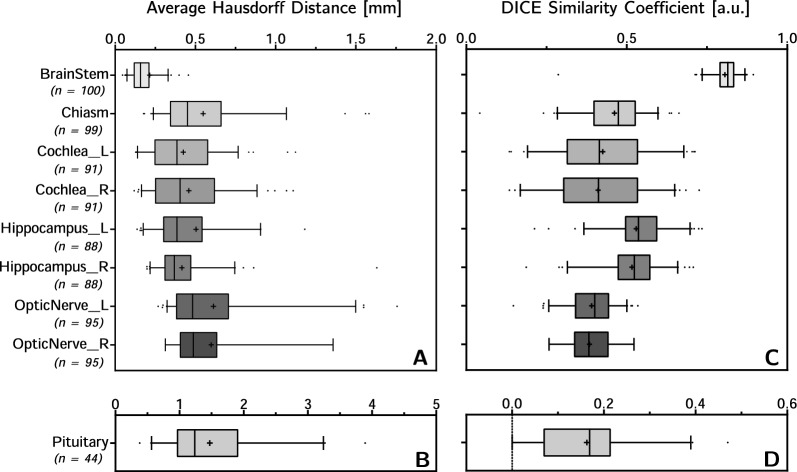
Average Hausdorff distance (**A** and **B**) and dice similarity coefficient (**C** and **D**) of OARs, Pituitary has its own graphs (**B** and **D**) the scale being too different from the other volumes. | represents the median. + represents the mean

The comparison between 100 CTVs shown in Fig. 4-A and B, yielded an average DSC (aDSC) value (5–95 percentile) of 0.94 (0.81$-$0.99) and average AHD (aAHD) value (5–95 percentile) of 0.04 mm (0.001$-$0.14 mm). Brain and bones had a aDSC of 0.95 (0.91$-$0.97) and 0.79 (0.72$-$0.84), and a aAHD of 0.04 mm (0.02$-$0.11 mm) and 0.29 mm (0.11$-$0.77 mm) respectively (Fig. 4C, D). By contrast, the aDSC and aAHD values for the OARs showed an overall wider range exemplified by the 5–95 percentile range as shown in Fig. 5 (see Appendix Table 3 for more detailed information). The brain stem (Fig. 5A, B) presented the highest average congruence values with aDSC of 0.81 (0.73$-$0.87) and aAHD of 0.21 mm (0.07$-$0.33 mm); while the pituitary gland (Fig. 5B, D) presented the lowest congruence values with aDSC of 0.17 (0$-$0.39) and aAHD of 1.37 mm (0.56$-$3.24 mm). It is important to note that the comparison of pituitary glands included only 44 patients as the structure was often not present in the CT-ss.

**Fig. 6:**
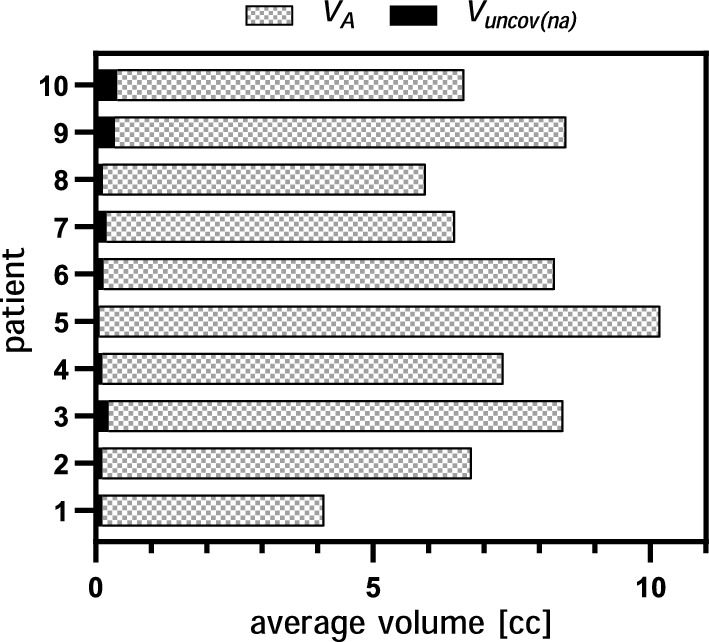
Comparison between the artefact volume (V$_{A}$) and the part that is left uncovered by PTV$_{na}$ (V$_{uncov(na)}$). For each patient, all volumes are averaged between the 5 CBCTs

Upon visual examination of all included sCT images (107 from the commissioning phase and 30 from the validation phase), artefacts were identified and quantified. Specifically, SFD artefacts were observed in 89 cases (65%), with type 1a being the most frequently encountered type, appearing in 194 instances (74%). Type 1b artefacts were the second most prevalent, observed in 56 instances (21%), followed by type 1c with the least occurrence at 11 instances (4%). Among the artefacts associated with dental implants, type 2a artefacts were the most common, appearing in 118 sCT images (86%), followed by type 2b artefacts with 10 instances (7%). Additionally, bone gaps were detected in 22 (16%) sCT images, with 10 of them exhibiting partial gaps and the remaining 12 (9%) displaying both partial and total gaps, affecting the entire width of the bone. Only 8 patients (6%) were observed completely free of any type of artefacts.

From the 20 type 1a SFD artefacts analysed in this study an average HD (5–95 percentile) of 3.33 mm (2.14$-$4.32 mm) was calculated. Therefore, it was suggested to extend the brain contour by approximately 3.5 mm beyond the bone for all patients with a SFD artefact within the target area during the validation phase.

The value of the V$_{uncov(a)}^{i}$, defined as the volume of the SFD artefact not covered by PTV$_{a}$ (equations 4), was equal to 0 in all but one patient, with a mean value (5–95 percentile) of 0.06 cc (0.05$-$0.06 cc). As for the value of V$_{uncov(na)}^{i}$, defined as the volume of the SFD artefact not covered by the new PTV$_{na}$ (equation 5), yielded a mean of 0.19 cc (0.07$-$0.40 cc). The average volumes for all CBCTs are shown in Fig. 6.

## Discussion

### Delineation

The purpose of the delineation comparison was to evaluate the reliability an MR-only workflow compared to current standard practice, in terms of target and organ-at-risk contouring.

The high congruence values (high DSC and low AHD) for the target seen in Fig. 4, suggest that there is no need for modification of contouring practices of the CTV. Likewise, the high congruence values found for the brain structure suggests that the modified procedure for brain contouring (e.g. use of Flood Fill tool instead of Segmentation Wizard tool) is equally reliable between sCT and CT.

As for the bone structure, it is worth noting the widespread values of DSC and AHD, despite the overall high congruence values observed between sCT and CT. This spread may be primarily caused by the mismatch of the spine between CT scans and sCT images due to patient movement that may occur despite fixation. Another contributing factor may be the observed differences in the sinus cavities as the interface between air and cortical bone is a known challenge for sCT generation [21, 22]. Nonetheless, the bone mean DSC obtained in this work, where the sCT image is AI-based, is in line with other reported data [23].

By contrast, OARs presented low congruence values as well as a substantial variability in DSC and AHD values, with the brain stem being the only exception, likely due to its ease of contouring and large volume. Inter-operator variablity and difference in voxel size between MR and CT, respectively sCT, may be two factors contributing to these findings. Indeed, our results are in line with other inter-operator studies reporting mean DSC values around 0.5 for optical nerves and below 0.4 for chiasm [24, 25]. Moreover, small structures (i.e., average volume below 1cc) like pituitary, chiasm, and cochleae are particularly susceptible to interpolation discrepancies when propagated by rigid registration between image modalities. Resampling of images to equal voxel size prior to deliniation would improve DSC and AHD values, however this procedure would not reflect the clinical practice.

### Artefacts

To the best of our knowledge, this is the first study that focuses on analysing the impact of sCT-artefacts caused by SFD on target contouring. Visual inspection of sCT images from 130 CNS patients led to a comprehensive classification of artefacts. This information was then mainly used for the development of the QA management program for MR-only workflow implementation. Thus, the staff could be trained for fast artefact identification and evaluation of sCT image usability. It is important to keep in mind that the artefact classification presented in this work may not be applicable to other sCT solutions due to variation in the training and validating data set, and the deep learning algorithm chosen [26, 27]. Regardless, the novel methodology described in this work offers simple and universal approach for analysing the impact that artefacts may have in the contouring process, and that can be applied on other sCT solutions. The added value of our results is that they can be utilised as benchmark for future software updates.

**Fig. 7:**
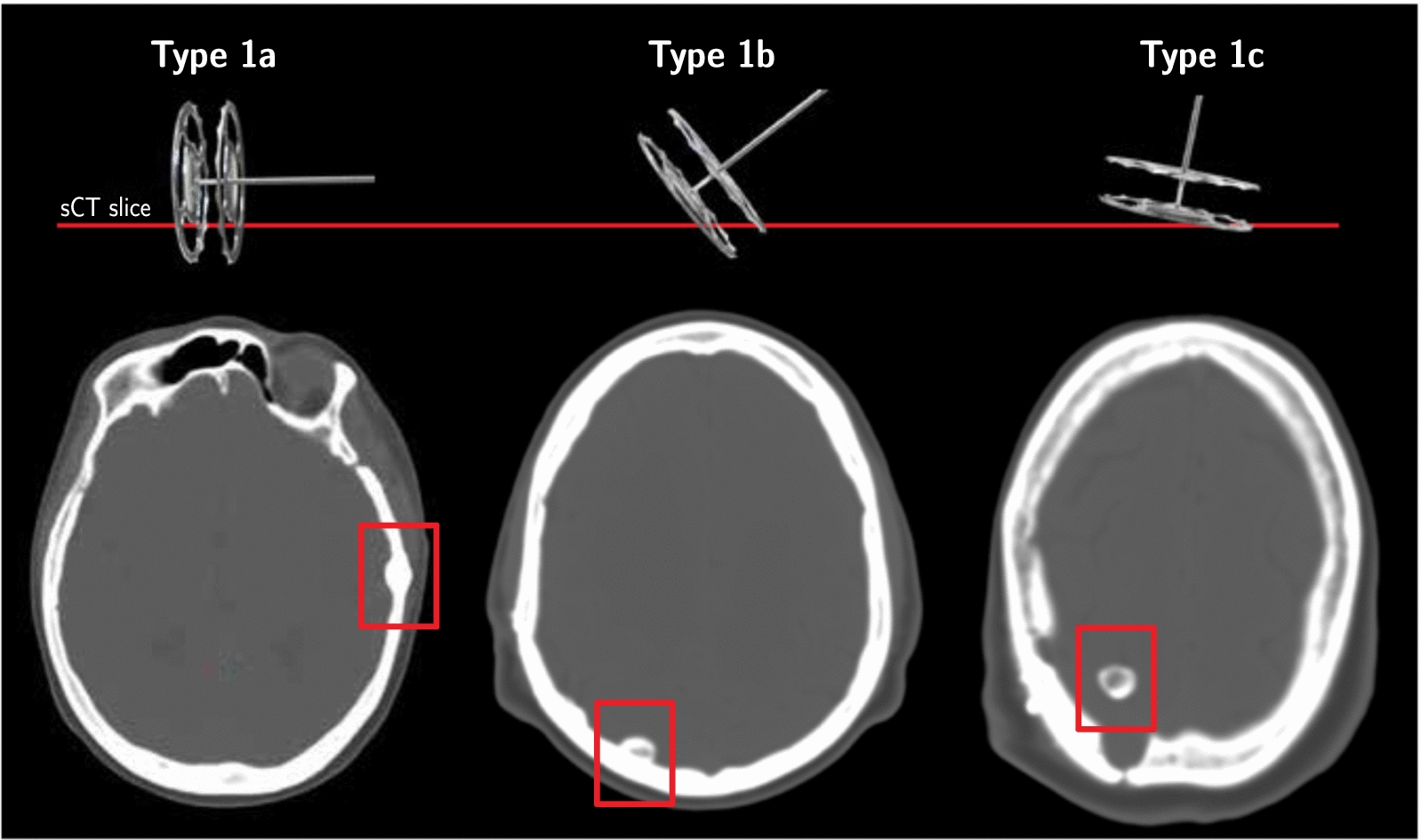
A 3D model of the surgical fixation device (SFD) is depicted at different angles with respect to the sCT slice. The red boxes inside the sCT image slice indicate the type of artefact generated due to the angle of the SFD

As pointed out by Rousselle et al. metal implants are a known problem when found within the target area [28]. In this study, SFDs were categorized based on their position and potential impact on target delineation (see Fig. 7). Although type 1c accounts for only 4% of all identified SFD artefacts, its distinct shape allows for easy manual detection and inclusion within the brain volume. On the other hand, type 1a and 1b, which make up 95% of all identified SFD artefacts, are prone to be overlooked and pose challenges in their precise delineation. Therefore this study focused on addressing them specifically.

Cropping the CTV to the automatically contoured brain on the sCT image results in a loss of volume coverage of up to 3.5 mm as suggested by our results. Therefore, during the validation phase a manual extension of the brain structure was performed and later evaluated to estimate the impact on dose coverage to the target.

For this, CBCT scans were chosen over CT scans despite being available for the validation cohort, for two reasons. Firstly, because on an MR-only workflow, the CBCT scan of the first treatment session is a candidate for performing sCT image QA [29]. Secondly, to assess inter-fraction variations. It is important to keep in mind that CBCT scans present beam hardening/streak artefacts due to metal implants [30] just as CT scans do. For this reason, visual comparison was performed between the CT and CBCT scans to ensure the same level of streak artefact between image modalities.

Our results demonstrated that the manual extension was effective and ensured coverage of the entire artefact volume. However, the manual adjustment of the brain introduced an additional step into the contouring process which has been found difficult to standardize and time consuming. Therefore, cropping the CTV to the bones instead of the brain was investigated as an alternative solution that would provide a simpler and more efficient contouring process in the presence of SFD artefacts. Our results show that, on average, only 2.8% of the artefact’s total volume (see Fig. 6) would be left uncovered by the standard PTV. Due to the palliative nature of glioblastoma treatment, the clinical impact that the CTV$_{na}$ may potentially miss these small volumes, corresponding to actual brain tissue, may be considered negligible. However, to implement an MR-only workflow for other curative CNS diagnosis or other treatment techniques such as stereotactic radiotherapy, a dosimetric analysis using, for example, isodose contours to evaluate coverage, is advised.

Type 3 artefacts or bone gaps, are rare occurrences as only 22 cases were identified across both commissioning (16 cases) and validation cohort (6 cases). The exact cause of these fake bone resection areas is unknown, despite increased accuracy in bone rendering from AI-based sCT algorithms [26]. The length of the artefacts found in the validation cohort ranged between 0.5 cm and 3 cm, with one exceptional case displaying a bone gap of 8 cm. A tolerance level of 3 cm has been adopted at our institutition which will be monitored in future patients, so that more appropriate adjustments can be made to the sCT image QA.

## Conclusions

In this study, we have investigated the feasibility of using a commercial sCT solution for radiotherapy of CNS tumors, focusing on the delineation of targets and the management of artefacts caused by SFDs. Our findings demonstrate that the tested sCT image can be used clinically in an MR-only workflow, as comparable target delineation accuracy to the CT based RT workflow was observed. The use of modern contouring atlases and evaluation of contouring performances among radio-oncologists further contributed to the validation of this workflow.

Furthermore, a method for determining the impact on delineation due to artefacts caused by surgical fixation devices described in this work, will hopefully encourage similar analysis for other commercially available sCT products for development of consensus QA criteria.

## Data Availability

The data supporting the findings of this study are not publicly available. However, they can be shared by the corresponding authors upon reasonable request.

## Appendix

See Table 3 and Fig. 7.

**Table 3:** Average of the congruency for each structure

Structure ID	n.	DSC (a.u.)	AHD (mm)	HD (mm)	Avg. vol. (cm^3^)
CTV	100	0.94 (0.81–0.99)	0.04 (0.001–0.14)	4,64 (0.68–9.27)	
Brain	100	0.95 (0.91–0.97)	0.04 (0.02–0.11)	10.52 (6.18–22.56)	1105.91
Bones	89	0.79 (0.72–0.84)	0.29 (0.11–0.77)	53.63 (15.47–109.2)	923.41
BrainStem	100	0.81 (0.73–0.87)	0.21 (0.07–0.33)	7.57 (3.95–11.43)	41.69
Chiasm	99	0.47 (0.28–0.60)	0.55 (0.24–1.01)	6.15 (2.36–12.56)	0.45
Cochlea_L	91	0.41 (0.19–0.68)	0.42 (0.14–0.77)	2.50 (1.21–4.26)	0.89
Cochlea_R	91	0.41 (0.17–0.65)	0.46 (0.14–0.77)	2.59 (1.21–4.52)	
Hippocampus_L	88	0.54 (0.37–0.70)	0.50 (0.17–0.91)	6.29 (2.98–11.12)	1.69
Hippocampus_R	88	0.52 (0.32–0.66)	0.42 (0.22–0.75)	6.21 (3.37–9.99)	
OpticNerve_L	95	0.40 (0.26–0.50)	0.61 (0.32–1.50)	6.12 (2.26–16.68)	0.88
OpticNerve_R	95	0.38 (0.26–0.52)	0.60 (0.31–1.36)	5.47 (2.67–12.63)	
Pituitary	44	0.17 (0–0.39)	1.47 (0.56–3.24)	6.11 (3.64–8.13)	0.28

Average volume has been calculated as one for the structures having both a left and a right side. The 5–95 percentile is given in parenthesis

*DSC* dice similarity coefficient, *AHD* average Hausdorff distance, *HD* Hausdorff distance

## Abbreviations

AHD: Average Hausdorff distance
AI: Artificial intelligence
CBCT: Cone beam computed tomography
CNS: Central Nervous System
CTV: Clinical target volume
CT: Computed tomography
DSC: Dice similarity coefficient
ED: Electron density
ESTRO: European Society for Radiotherapy and Oncology
EPTN: European Particle Therapy Network
FLAIR: Fluid-attenuated inversion recovery
FFE: Fast field echo
HD: Hausdorff distance
HU: Hounsfield units
MRCAT: Magnetic Resonance for Calculating Attenuation Map
MRI T1 Gd+: T1-weighted with contrast medium Gadolinium
ML: Machine learning
MR: Magnetic resonance
OARs: Organs at risk
PTV: Planning target volume
QA: Quality assurance
RT: Radiotherapy
sCT: Synthetic CT
SFD: Surgical fixation device
ss: Structure set
TPS: Treatment planning system
WHO: World Health Organization
